# Paucity of CD4^+^ Natural Killer T (NKT) Lymphocytes in Sooty Mangabeys Is Associated with Lack of NKT Cell Depletion after SIV Infection

**DOI:** 10.1371/journal.pone.0009787

**Published:** 2010-03-24

**Authors:** Namita Rout, James G. Else, Simon Yue, Michelle Connole, Mark A. Exley, Amitinder Kaur

**Affiliations:** 1 New England Primate Research Center, Harvard Medical School, Southborough, Massachusetts, United States of America; 2 Yerkes National Primate Research Center, Emory University, Atlanta, Georgia, United States of America; 3 Beth Israel Deaconess Medical Center, Harvard Medical School, Boston, Massachusetts, United States of America; University of California San Francisco, United States of America

## Abstract

Lack of chronic immune activation in the presence of persistent viremia is a key feature that distinguishes nonpathogenic simian immunodeficiency virus (SIV) infection in natural hosts from pathogenic SIV and HIV infection. To elucidate novel mechanisms downmodulating immune activation in natural hosts of SIV infection, we investigated natural killer T (NKT) lymphocytes in sooty mangabeys. NKT lymphocytes are a potent immunoregulatory arm of the innate immune system that recognize glycolipid antigens presented on the nonpolymorphic MHC-class I-like CD1d molecules. In a cross-sectional analysis of 50 SIV-negative and 50 naturally SIV-infected sooty mangabeys, ligand α-galactosylceramide loaded CD1d tetramers co-staining with Vα24-positive invariant NKT lymphocytes were detected at frequencies ≥0.002% of circulating T lymphocytes in approximately half of the animals. In contrast to published reports in Asian macaques, sooty mangabey NKT lymphocytes consisted of CD8^+^ and CD4/CD8 double-negative T lymphocytes that were CXCR3-positive and CCR5-negative suggesting that they trafficked to sites of inflammation without being susceptible to SIV infection. Consistent with these findings, there was no difference in the frequency or phenotype of NKT lymphocytes between SIV-negative and SIV-infected sooty mangabeys. On stimulation with α-galactosylceramide loaded on human CD1d molecules, sooty mangabey NKT lymphocytes underwent degranulation and secreted IFN-γ, TNF-α, IL-2, IL-13, and IL-10, indicating the presence of both effector and immunoregulatory functional capabilities. The unique absence of CD4^+^ NKT lymphocytes in sooty mangabeys, combined with their IL-10 cytokine-secreting ability and preservation following SIV infection, raises the possibility that NKT lymphocytes might play a role in downmodulating immune activation in SIV-infected sooty mangabeys.

## Introduction

While persistent immune activation is a strong prognosticator of disease progression in HIV-infected humans and SIV-infected Asian macaques, it is singularly lacking in non-progressive infection in natural hosts of SIV such as sooty mangabeys and African green monkeys [1], [2], [3], [4]. How natural hosts of SIV are able to contain chronic immune activation in the face of continuing viral replication and high viral loads remains a conundrum [5]. Immune activation is observed during acute SIV infection in sooty mangabeys and African green monkeys, but it is rapidly down-regulated to pre-SIV infection levels [6], [7], [8], [9], [10]. Mechanisms that have been implicated in down-modulation of immune activation in natural hosts include early induction of an anti-inflammatory response [11], absence of microbial translocation [12], paucity of CCR5^+^ CD4^+^ T lymphocytes [13], decreased responsiveness of plasmacytoid dendritic cells to SIV [14], and preservation of Th17 CD4^+^ T lymphocytes [15], [16]. The magnitude of SIV-specific T lymphocyte responses in sooty mangabeys during acute and chronic SIV infection is comparable to that in rhesus macaques [9], [17], [18] and hence, differences in the adaptive immune response to SIV are unlikely to be responsible for the differential immune activation of pathogenic and nonpathogenic SIV infection. The rapid activation of multiple pro-inflammatory cytokines and chemokines in acute HIV and SIV infection point to the early innate immune response as an important determinant of immune activation [9], [19].

Natural Killer T (NKT) lymphocytes are a small subset of T lymphocytes that are rapid responders of the innate immune system and mediate potent immunoregulatory and effector functions in a variety of disease settings [20], [21]. NKT lymphocytes recognize antigen presented by the non-polymorphic MHC Class-I-like CD1d molecules and are characterized by a restricted TCR repertoire due to the presence of an invariant TCR alpha chain paired with a limited number of TCR beta chains [20], [21]. In mice, the TCR of invariant NKT lymphocytes consists of a Vα14-Jα18 chain paired with Vβ8.2, Vβ7 or Vβ2, while human invariant NKT lymphocytes have a Vα24-Jα18 chain preferentially paired with Vβ11 [22], [23], [24]. Upon activation with glycolipids presented on CD1d molecules, NKT lymphocytes respond rapidly with production of a diverse array of cytokines including IL-10, TGF-β, and several Th1 and Th2 cytokines [25], [26]. Studies in mice suggest that the NKT lymphocytes transcribe cytokine genes even before activation and are therefore able to respond rapidly upon TCR stimulation [27]. Because NKT lymphocytes can produce a wide array of cytokines without the requirement of priming, they can modulate other arms of the innate and adaptive immune system and mediate diverse, often polar functions [28], [29], [30], [31]. Thus, while NKT lymphocytes can mediate anti-tumor [32] and anti-microbial effector activity against selected pathogens [33], [34], [35], [36], [37], [38], they also induce tolerance and play a predominantly immunoregulatory role in corneal graft tolerance, inhibition of autoimmune diabetes in NOD mice, and regulation of immunopathology in murine *Mycobacterium bovis* and lymphocytic choriomeningitis virus infections [37], [39], [40], [41], [42].

The precise role of NKT lymphocytes in HIV/SIV infection remains unresolved. CD4^+^ NKT lymphocytes in humans and macaques express CCR5 and are highly susceptible to HIV/SIV infection *in vitro* [43], [44], [45]. A selective and rapid loss of both CD4^+^ and CD4^−^ NKT lymphocytes has been observed in the peripheral blood of HIV-infected humans [43], [46], [47], [48] with variable reconstitution after anti-retroviral therapy [49], [50], [51], [52], [53]. A recent study showed a similar decline of CD4^+^ NKT lymphocytes in SIV-infected pig-tailed macaques [54]. In addition to preferential infection of CD4^+^ NKT lymphocytes by R5-tropic HIV and SIV, Fas-mediated activation-induced apoptosis and tissue sequestration have been implicated as possible causal mechanisms of NKT lymphocyte depletion in pathogenic HIV/SIV infection [43], [45], [48], [49]. Although the consequences of NKT lymphocyte depletion in HIV infection are not known, it is speculated that loss of NKT lymphocytes might result in increased susceptibility to opportunistic infections as well as contribute to increased aberrant immune activation [48], [52].

In light of the immunoregulatory properties of NKT lymphocytes and their potential to down-regulate immune activation, we have investigated NKT lymphocytes in a natural host of SIV infection. In this study, we report on a detailed characterization of NKT lymphocytes in sooty mangabeys. We show that in contrast to published reports of NKT lymphocytes in Asian macaques, CD1d-restricted invariant NKT lymphocytes in sooty mangabeys do not express CD4 or CCR5. In a cross-sectional analysis, naturally SIV-infected sooty mangabeys had similar frequency and phenotype of circulating NKT lymphocytes compared to SIV-negative sooty mangabeys. Functionally, sooty mangabey NKT lymphocytes showed robust production of Th1 and Th2 cytokines, including IL-10, and underwent degranulation on NKT ligand-specific activation indicating that they had both effector and immunoregulatory functional capabilities. The IL-10 cytokine-secreting profile of sooty mangabey NKT lymphocytes along with their preservation in naturally SIV-infected mangabeys raises the possibility that NKT lymphocytes play an important role in down-regulating immune activation during natural SIV infection.

## Results

### Identification of NKT lymphocytes in sooty mangabeys

**Figure 1:**
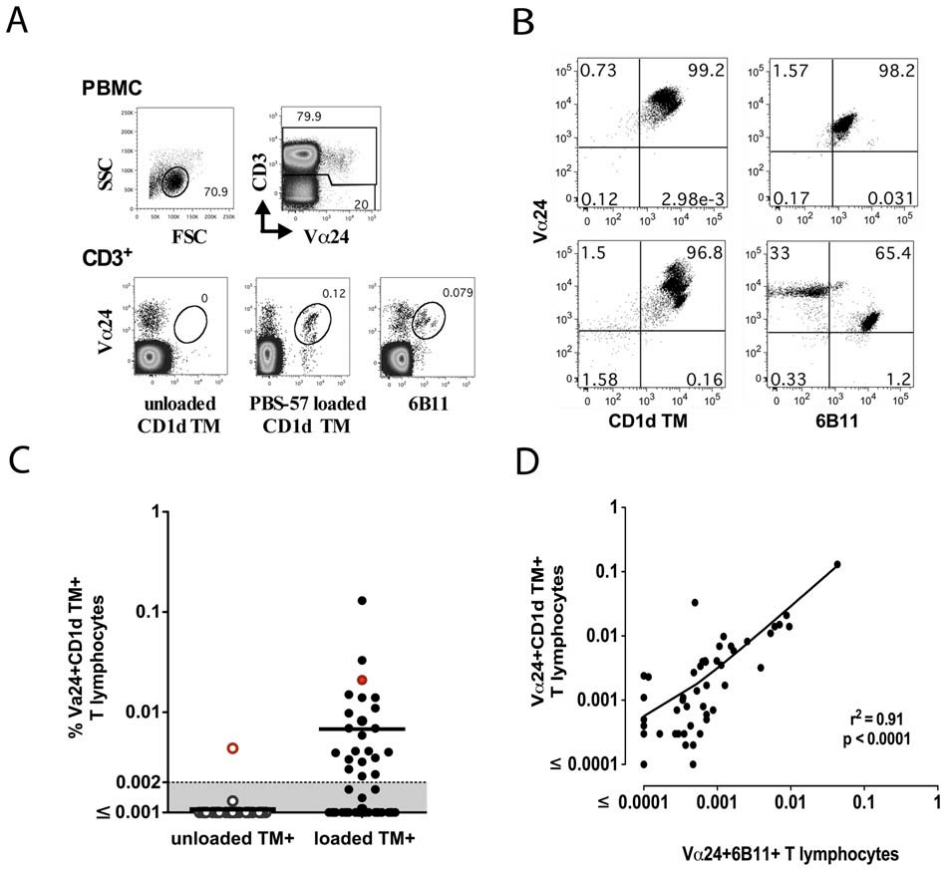
Identification of NKT lymphocytes in the peripheral blood of sooty mangabeys. A) Representative dot plots showing gating strategy for identification of NKT lymphocytes. On the CD3^+^ T lymphocyte population, the co-staining for Vα24 and CD1d tetramers loaded with PBS-57 (CD1d TM) and 6B11 are shown in the bottom panel. Unloaded tetramers (shown in the left of bottom panel) served as a control for non-specific staining. B) Representative dot plots of sorted NKT clones in one SIV-negative sooty mangabey stained with anti-Vα24 and CD1d TM or 6B11 antibody. C) Frequency of peripheral blood NKT lymphocytes in 50 SIV-negative sooty mangabeys. Horizontal bar denotes mean. The background staining with unloaded CD1d TM for one animal (which was above 0.002%) and the corresponding Vα24^+^CD1d TM^+^ frequency are shown in red. D) Positive correlation between the frequency of Vα24^+^CD1d TM^+^ T lymphocytes and Vα24^+^6B11^+^ T lymphocytes in the peripheral blood of 50 SIV-negative sooty mangabeys.

The majority of human CD1d-reactive NKT lymphocytes express the invariant Vα24-Jα18 TCR α-chain paired with the Vβ11 TCR β-chain and are identified by flow cytometric detection of Vα24 TCR-positive T lymphocytes that either bind to the 6B11 mAb directed against the invariant CDR3 region of the TCR α-chain [55], or that bind to human CD1d tetramers loaded with ligand α-galactosylceramide (α-GalCer) or its analog, PBS-57 [56], [57]. Using a similar approach in sooty mangabeys, staining of PBMC with anti-human Vα24 antibody and PBS-57 loaded CD1d tetramers (CD1d TM) or the 6B11 antibody, revealed a small but discrete population of Vα24^+^CD1d TM^+^ and Vα24^+^6B11^+^ NKT lymphocytes (Figure 1A). Limiting dilution cloning of sorted NKT lymphocytes in one SIV-negative sooty mangabey revealed that 97% of the NKT clones were concurrently Vα24^+^ and 6B11^+^ (Figure 1B top panel) confirming their invariant nature. A small subset of Vα24^+^CD1d TM^+^ cells were not 6B11^+^ (Figure 1B bottom panel), indicating the presence of a minor population of non-invariant NKT lymphocytes in sooty mangabeys, as have been reported in humans [58], [59], [60].

In a cross-sectional analysis of fifty SIV-negative sooty mangabeys, Vα24^+^CD1d TM^+^ NKT lymphocytes ranged in frequencies from 0 to 0.13% of circulating T lymphocytes in the peripheral blood (Figure 1C). Owing to the rarity of circulating NKT lymphocytes, a minimum of 200,000 CD3^+^ T lymphocyte events were collected to ensure that detection of NKT lymphocytes at frequencies less than 0.01% reached a power of ≥80% at a *P* value <0.05. Additionally, the concurrent use of unloaded CD1d TM provided a valuable negative control for specificity of staining with PBS-57-loaded CD1d TM (Figure 1C). With the exception of one mangabey, the background staining with unloaded CD1d TM was well below 0.002% (Figure. 1C). Using an arbitrary positive cut-off value of ≥0.002%, *ex vivo* circulating NKT lymphocytes were detected at a mean frequency of 0.007% in 44% of the SIV-negative sooty mangabeys studied (Figure 1C). The frequency of NKT lymphocytes detected by the 6B11 antibody tended to be lower compared to detection with CD1d TM. However, there was a highly significant positive correlation between the frequency of peripheral blood Vα24^+^CD1d TM^+^ and Vα24^+^6B11^+^ NKT lymphocytes (Figure 1D), confirming a dominance of invariant NKT lymphocytes in the CD1d TM^+^ T lymphocyte population.

### NKT lymphocytes in sooty mangabeys are comprised of CD8^+^ and CD/CD8 double negative (DN) T lymphocytes

**Figure 2:**
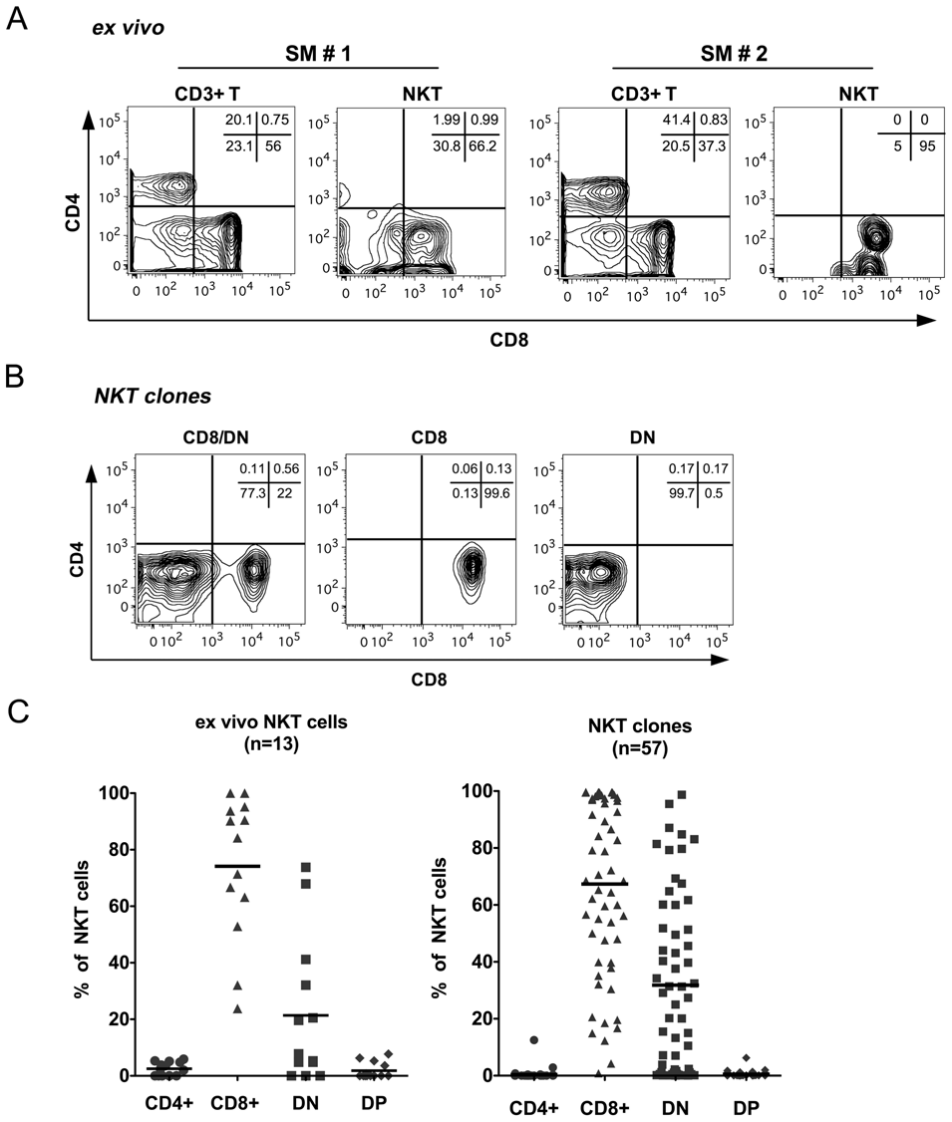
T lymphocyte subset distribution of sooty mangabey NKT lymphocytes. A) Representative contour plots showing surface expression of CD4 and CD8 molecules on *ex vivo* circulating T and Vα24^+^CD1d TM^+^ NKT lymphocytes in two sooty mangabeys (SM #1 and SM #2). B) Contour plots showing surface expression pattern of CD4 and CD8 molecules on representative NKT clones. C) Distribution of CD4^+^ (CD4^+^8^−^), CD8^+^ (CD8^+^4^−^), DN (CD4^−^8^−^) and DP (CD4^+^8^+^) T lymphocyte subsets in *ex vivo* NKT lymphocytes in 13 SIV-negative sooty mangabeys (left panel) and in 57 NKT clones derived from sorted 6B11^+^ T lymphocytes of one SIV-negative sooty mangabey (right panel).

While most human and essentially all murine NKT lymphocytes consist of CD4^+^ and DN T lymphocyte subsets [24], [61], published reports in Asian macaques have shown the presence of predominantly CD8^+^ and CD4^+^ NKT lymphocyte subsets [45], [54], [62], [63]. Evaluation of CD4 and CD8 surface expression on *ex vivo* circulating NKT lymphocytes in 13 SIV-negative sooty mangabeys, revealed the presence of both CD8^+^ (mean ± SD; 79.3%±22.6) and DN (17.5%±21.6) subsets of NKT lymphocytes (Figure 2A & 2C left panel). In contrast to Asian macaques, CD4^+^ NKT lymphocytes were absent or detected at frequencies <5% of NKT cells in sooty mangabeys. Consistent with their phenotype, sooty mangabey NKT lymphocytes were significantly enriched for CD8^+^ T lymphocytes and had significantly lower number of CD4^+^ T lymphocytes compared to the circulating total T lymphocyte population (Table 1). The frequency of NKT lymphocytes within circulating CD8^+^ T lymphocytes ranged between 0% and 0.13% (mean 0.008%), while DN T lymphocytes contained 0% to 0.16% (mean 0.007%) of NKT lymphocytes.

**Table 1:** Distribution of T lymphocyte subsets in circulating NKT cells and total T lymphocytes in sooty mangabeys.

			Mean % ± SD@	
	CD4^+^	CD8^+^	CD4^−^CD8^−^	CD4^+^CD8^+^
**CD3** ^+^ **T cells**	35±7	45±7	19±4	0.5±0.4
**NKT cells**	2.5±2.3	74±25	21±25	1.8±2.8
**P-Value** *	<0.0001	<0.001	0.79	0.13

*Paired *t*-test.

^@^Mean % and Standard Deviation in 13 SIV-negative sooty mangabeys.

The CD4 and CD8 subset distribution of 57 NKT clones isolated from one SIV-negative sooty mangabey were similar to that of the *ex vivo* NKT lymphocytes (Figure 2B & 2C right panel). In addition to a mixed CD8^+^ and DN T lymphocyte phenotype, there were also a substantial number of clones that displayed a predominant or exclusive CD8^+^ or DN T lymphocyte phenotype (Figure 2C). Further analysis of CD8 expression using CD8α and CD8β antibodies revealed that NKT lymphocytes expressed CD8αα homodimers (data not shown). Consistent with findings on *ex vivo* PBMC, none of the NKT clones had a CD4^+^ T lymphocyte phenotype (Figure 2C).

### Phenotypic characterization of sooty mangabey NKT lymphocytes

**Figure 3:**
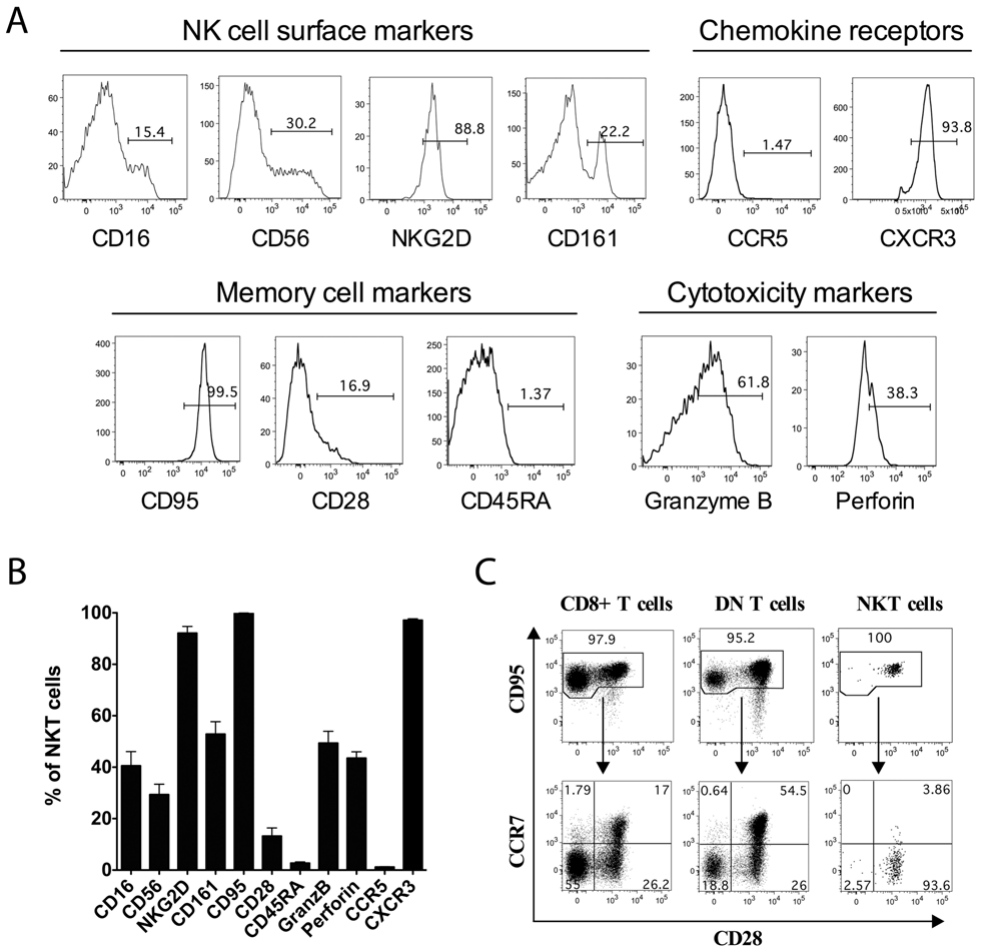
Phenotypic characterization of sooty mangabey NKT clones. A) Histogram plots gated on representative NKT clones showing surface expression of NK cell markers (CD16, CD56, NKG2D, and CD161), chemokine receptors (CCR5 and CXCR3), and memory markers (CD95, CD28, and CD45RA), and intracellular expression of cytolytic molecules (granzyme B and perforin). B) Mean % for each phenotypic marker on 5 to 71 NKT clones. Error bars denote standard error of mean (SEM). C) Memory phenotype of NKT lymphocytes in comparison to CD8^+^ and DN T lymphocytes in the peripheral blood of one SIV-negative sooty mangabey. Dot plots showing CD95 vs CD28 staining on each subset of T lymphocytes (top panel) and distribution of CCR7 and CD28 on the CD95^+^ memory lymphocytes (bottom panel).

To investigate the properties of sooty mangabey NKT lymphocytes, we first examined the expression of NK cell markers, memory T lymphocyte markers and cytolytic molecules on sooty mangabey NKT clones (Figure 3A–B). Human and mouse NKT lymphocytes express the NK cell markers CD161A [64] and CD161C/NK1.1 respectively [65], while macaque NKT lymphocytes from the spleen were reported to express CD56 [63]. We examined the expression of the NK cell markers CD56, CD161, CD16, and NKG2D on sooty mangabey NKT clones. While the majority of mangabey NKT clones (92.1%±17.2) expressed NKG2D, there was considerable heterogeneity in the expression of CD161 (52.8%±32.6), CD16 (40.5%±36.6) and CD56 (29.3%±26.9%) within clones (Figure 3B). Analysis of the surface markers CD95, CD28, CD45RA, and CXCR3 showed that the majority of the NKT clones were CD95^+^CD28^−^CD45RA^−^ and CXCR3^+^ (Figure 3A–B) indicating an effector memory phenotype consistent with findings in human NKT lymphocytes [43], [66]. In addition, the NKT clones contained intracellular granzyme B (49.3%±38.7) and perforin (43.5%±20) suggesting the potential for cytolytic activity (Figure 3A–B). This is in concordance with published reports of cytolytic activity in human NKT lymphocytes [61], [64], [67], [68].

Since the initial phenotypic characterization was performed on *in vitro* cultured NKT clones, we asked whether *ex vivo* circulating NKT lymphocytes displayed a similar phenotype. Peripheral blood NKT lymphocytes from one SIV-negative sooty mangabey from whom the NKT clones were derived showed a similar phenotype to clones with regards to a high frequency of CD95, NKG2D and CXCR3 surface expression (Table 2). However, in contrast to NKT clones, peripheral blood NKT lymphocytes were predominantly CD28^+^ (Table 2 and Figure 3C). This is consistent with loss of CD28 expression on tissue cultured human NKT lymphocytes [64], [67] as opposed to *ex vivo* human NKT lymphocytes [61], [68]. *Ex vivo* NKT lymphocytes also differed from NKT clones in having a paucity of cells expressing the NK markers CD56 and CD161, and the cytolytic molecules granzyme B and perforin (Table 2). In comparison to memory CD8^+^ and DN T lymphocytes, *ex vivo* NKT lymphocytes consisted of a homogenous CD95^+^CD28^+^CCR7^−^ population, consistent with a transitional effector memory phenotype (Fig. 3C). The phenotypic differences between *ex vivo* NKT lymphocytes and NKT clones is likely a reflection of the activated status of NKT clones subsequent to *in vitro* stimulation with α-GalCer and IL-2.

**Table 2:** Phenotypic comparison of NKT clones and *ex vivo* circulating NKT lymphocytes from one SIV-negative sooty mangabey.

	% positive NKT lymphocytes	
	*ex vivo*	clones*
CD95^+^CD28^+^	96.8	13.9
CD95^+^CD28^−^	3.2	85.7
CXCR3^+^	95.4	97.1
CCR5^+^	ND	1.2
CD16^+^CD56^+^	0	10.4
CD16^+^CD56^−^	11.7	0.2
CD16^−^CD56^+^	1.4	56
CD161^+^	8.5	59.6
NKG2D^+^	75	92
NKG2A^+^	10.3	ND
Granzyme B^+^	2.3	49.3
Perforin^+^	1.7	43.4

*Mean % of 27 clones.

ND- not determined.

### Sooty mangabey NKT clones secrete Th1 and Th2 cytokines, degranulate, and proliferate in a CD1d-restricted manner

**Figure 4:**
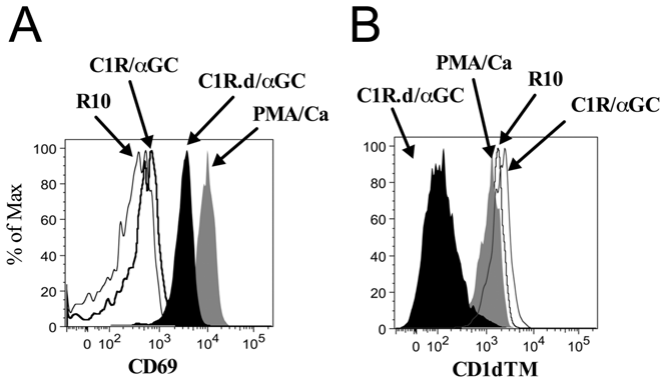
Activation and TCR downregulation in sooty mangabey NKT clones. Representative histogram plots on one NKT cell clone showing (A) surface expression of CD69 and (B) TCR detection by CD1d TM following 16 hours *in vitro* stimulation with the NKT-specific ligand α-GalCer presented on C1R cell lines expressing CD1d (C1R.d/α-GC), C1R cell lines pulsed with α-GalCer (C1R/α-GC), PMA/Ca or medium alone (R10).

To investigate the functionality of sooty mangabey NKT lymphocytes, we first investigated the cytokine-secreting profile of NKT clones following stimulation with CD1d-transfected cell lines pulsed with α-GalCer (C1R.d/αGC). Stimulation with mock-transfected C1R cell lines pulsed with α-GalCer (C1R/αGC) served as a negative control. *In vitro* stimulation of NKT clones with either mitogen (PMA/Ca) or C1R.d/αGC resulted in up-regulation of CD69 indicating NKT cell activation (Figure 4A). There was a concomitant decrease in CD1d TM staining that was confined to the C1R.d/αGC stimulated lymphocytes (Figure 4B) indicating that the NKT lymphocyte TCR was down-regulated following ligand-specific stimulation.

**Figure 5:**
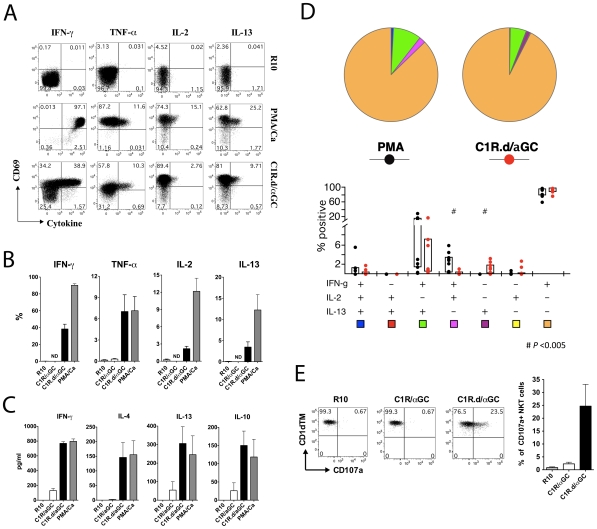
Th1 and Th2 cytokine production by sooty mangabey NKT clones. A) Intracellular cytokine staining of one representative NKT clone with antibodies against IFN-γ, TNF-α, IL-2, and IL-13 following 16 hours *in vitro* stimulation with PMA/Ca or the specific ligand α-GalCer presented on C1R.d cells (C1R.d/α-GC). Medium alone (R10) served as a negative control. Dot plots gated on NKT lymphocytes show intracellular expression of recent activation marker CD69 versus each cytokine with stimulation conditions denoted on the right of each panel. Numbers denote the % of NKT cells in each quadrant. B) Mean frequency of cytokine-positive NKT cells. Data on 9 NKT clones shown. Error bars denote SEM. ND: Not determined. C) Cytokine ELISA for IFN-γ, IL-4, IL-13, and IL-10 on culture supernatants collected at 24 hours post-stimulation. Mean and SEM of four clones shown. D) Cytokine profile of the NKT clones with regards to the concurrent production of IFN-γ, IL-2, and IL-13. Data on 9 NKT clones after 16 hours of stimulation shown. Pie charts (top panel) showing the proportion of one-, two-, and three-functional responses in cytokine-secreting 6B11^+^ cells. Bar charts (bottom panel) showing the mean proportion of responding cells for each of seven functional combinations. Boxes represent interquartile ranges. E) Representative dot plots (on the left) gated on NKT clones showing CD107a surface expression in CD1d/α-GalCer stimulated NKT lymphocytes in comparison to cells stimulated with C1R/α-GalCer or medium alone for 4 hours. Mean frequency of NKT clones degranulating after 4-hour stimulation (on the right). Data on surface upregulation of CD107a on four NKT cell clones shown. Error bars denote SEM.

At the end of a 16 to 24-hour stimulation period with C1R.d/αGC, production of the Th1 cytokines IFN-γ, TNF-α, and IL-2, and the Th2 cytokines IL-4, IL-13, and IL-10, was observed in NKT clones by intra-cellular cytokine flow cytometry (Figure 5A–B) and ELISA (Figure 5C). Interestingly, IL-17 production was not detected (data not shown). This contrasts with a recent report on emergence of IL-17 producing NKT lymphocytes in SIV-infected rhesus macaques [69]. Cytokine production following C1R.d/αGC stimulation was close to levels induced after PMA/Ca stimulation (Figure 5B–C) indicating the potency of NKT ligand-specific stimulation for activation of NKT clones. In contrast, stimulation with control C1R/αGC induced little or no cytokine production (Figure 5B–C). To determine whether both Th1 and Th2 cytokines were produced by the same NKT cell, we investigated concurrent production of IFN-γ, IL-2, and IL-13 by NKT clones stimulated with C1R.d/αGC and PMA/Ca (Figure 5D). More than 60% of cytokine-secreting NKT cells secreted only IFN-γ after C1R.d/αGC or PMA/Ca stimulation (Figure 5D). Concurrent production of IFN-γ and IL-13 was observed in <20% of cytokine-secreting NKT cells (Figure 5D). Comparison of mitogen stimulation with that of NKT ligand-specific stimulation showed significant differences with regards to cytokine production. Concurrent IFN-γ and IL-2 production in the absence of IL-13 was only seen after PMA/Ca but not C1R.d/αGC stimulation (Figure 5D). Furthermore, production of IL-13 alone in the absence of the Th1 cytokines IFN-γ and IL-2 was only seen after C1R.d/αGC stimulation (Figure 5D), suggesting the presence of discrete Th2 cytokine-secreting NKT lymphocyte subsets.

**Figure 6:**
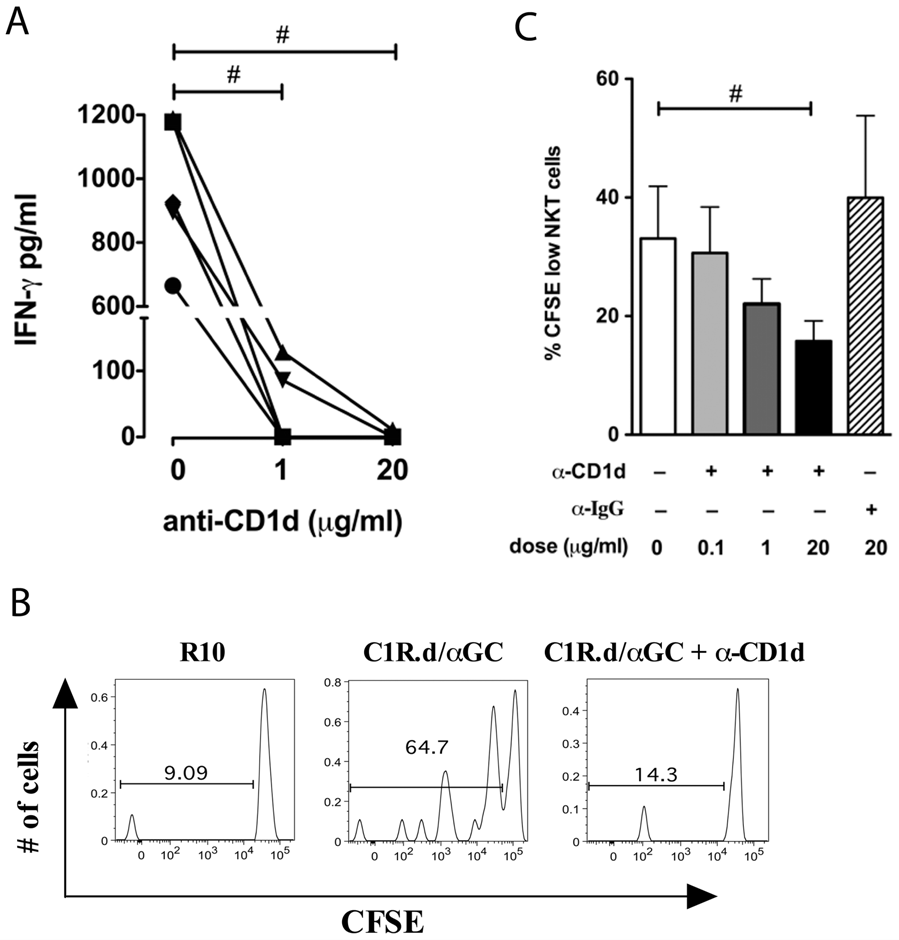
Sooty mangabey NKT lymphocytes respond to NKT ligands in a CD1d-restricted manner. A) IFN-γ ELISA on culture supernatants of NKT clones stimulated with C1R.d/α-GalCer (50 ng/ml) for 16 hours in the presence of anti-CD1d antibody (clone 42.1) at 0, 1, and 20 µg/ml. Data shows IFN- γ secretion from five clones. B) Representative histogram plots gated on NKT lymphocytes showing proliferation assessed by CFSE staining after 5 days of CD1d/α-GalCer stimulation in the presence or absence of anti-human CD1d mAb (clone 42.1). C) Dose-dependent reduction in proliferation of NKT cells with anti-CD1d. Mean and SEM of five NKT clones shown.

In addition to cytokine production, NKT clones also underwent degranulation upon stimulation with C1R.d/αGC, as evidenced by the surface expression of CD107a (Figure 5E). The degranulation was evident as early as four hours following stimulation, suggesting that sooty mangabey NKT clones were capable of rapid cytolytic activity. The CD1d-restricted nature of NKT lymphocyte activation was confirmed by partial to complete inhibition of cytokine secretion and proliferation in the presence of anti-CD1d blocking antibody (Figure 6). Addition of anti-human CD1d mAb (clone 42.1) to C1R.d cells prior to pulsing with α-GalCer, inhibited IFN-γ production by C1R.d/αGC-stimulated NKT clones in a dose-dependent manner (Figure 6A). Functional CD1d restriction of sooty mangabey NKT clones was further verified by examination of α-GalCer-induced proliferation. Proliferation of CFSE-labeled NKT clones was observed after a 5-day period of stimulation with C1R.d/αGC (Figure 6B). The proliferative response to C1R.d/αGC was significantly inhibited in the responding NKT clones treated with anti-CD1d in a dose-dependent manner (Figure 6B–C). No inhibition of proliferation was observed with control isotype Ab (Figure 6C).

### Cytokine responses of ex vivo peripheral blood NKT lymphocytes in sooty mangabeys mirror those of NKT clones

**Figure 7:**
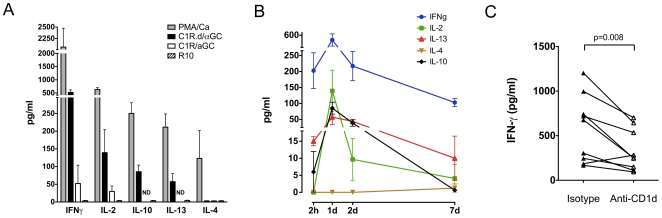
Cytokine production by *ex vivo* peripheral blood NKT lymphocytes in sooty mangabeys. A) Cytokine ELISA for IFN-γ, IL-2, IL-10, IL-13, and IL-4 with culture supernatants collected 24 hours post-stimulation of PBMC (mean and SEM of 4 to 7 animals). B) Kinetics of cytokine secretion in PMA/Ca and C1R.d/α-GC stimulated PBMCs at 2 hour, 1 day, 2 days, and 7 days post-stimulation. C) IFN-γ production in C1R.d/α-GC stimulated lymphocytes in the presence of anti-human CD1d (clone 42.1) blocking antibody or isotype control Ab.

To determine whether the functional profile of *in vitro* expanded NKT clones reflected the properties of circulating NKT lymphocytes, we analyzed the cytokine secretion of *ex vivo* NKT lymphocytes in seven SIV-negative sooty mangabeys. The cytokine response of *ex vivo* peripheral blood NKT lymphocytes following NKT ligand-specific stimulation was assessed by ELISA. After a 24-hour stimulation period, supernatants of PBMC cultured with C1R.d/αGC showed production of IFN-γ, IL-2, IL-10, and IL-13 (Figure 7A). Stimulation with α-GalCer in the absence of CD1d (C1R/αGC) also resulted in low levels of IFN-γ and IL-2 production (Figure 7A). This may either reflect presentation of soluble α-GalCer by CD1d-positive APCs present in PBMC or a xenogenic response of mangabey PBMC to human-derived C1R cells. With the exception of IL-4, the cytokine secreting profile C1R.d/αGC-stimulated PBMC was similar to that of NKT clones. IL-4 was not detected in culture supernatants of PBMC stimulated with C1R.d/αGC, but was detected after PMA/Ca stimulation. A kinetic analysis of cytokine production over a 2 hour to 7-day period also failed to detect any IL-4 production after C1R.d/αGC stimulation (Figure 7B). IFN-γ was the dominant cytokine response, starting as early as 2 hours and peaking at one day (Figure 7B). In addition to IFN-γ, low levels of IL-13 and IL-10 were also detected at 2 hours. Similar to IFN-γ, production of IL-2, IL-13, and IL-10 peaked at 24 hours (Figure 7B).

In order to verify the specificity of the cytokine response on C1R.d/αGC stimulation, PBMC were stimulated in the presence of anti-CD1d blocking antibody. There was a significant 1.3-fold to 3-fold reduction in IFN-γ production in PBMC treated with anti-CD1d antibody in comparison to untreated PBMC (Figure 7C), indicating that the cytokine response on C1R.d/αGC stimulation PBMC was a result of NKT lymphocyte stimulation. There was no correlation between the *ex vivo* NKT lymphocyte frequencies and the amount of cytokine production (data not shown).

### Peripheral blood NKT lymphocytes are not depleted in SIV-infected sooty mangabeys

**Figure 8:**
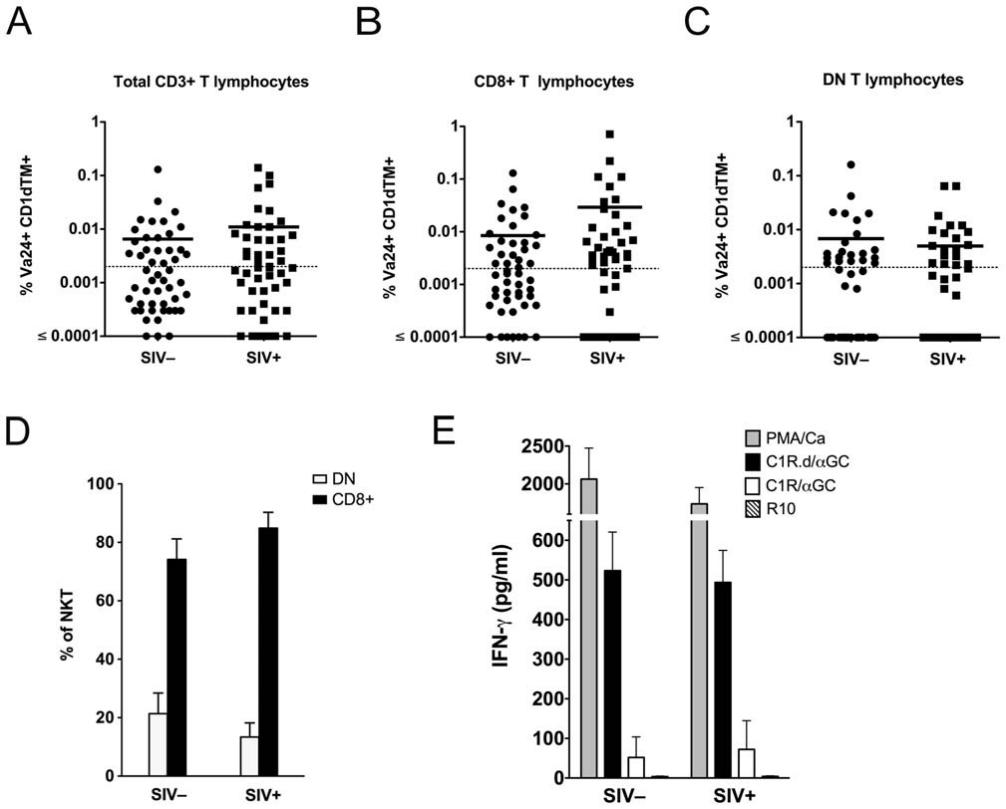
NKT lymphocytes are not depleted in SIV-infected sooty mangabeys. Frequency of peripheral blood Vα24^+^CD1d TM^+^ NKT lymphocytes in 50 SIV-negative and 50 SIV-infected sooty mangabeys as a percentage of (A) total circulating T lymphocytes, (B) circulating CD8^+^ T lymphocytes, and (C) circulating DN T lymphocytes. Horizontal bar denotes mean. D) Percentage of CD8+ and DN T lymphocyte subsets in peripheral blood NKT lymphocytes in SIV− and SIV+ sooty mangabeys. E) IFN-γ ELISA on 24-hour culture supernatants of ex vivo PBMC stimulated with PMA/Ca, C1R.d/α-GC, C1R/α-GC, or medium alone (R10). Mean and SEM of data on six SIV-negative and six SIV-infected sooty mangabeys shown.

**Figure 9:**
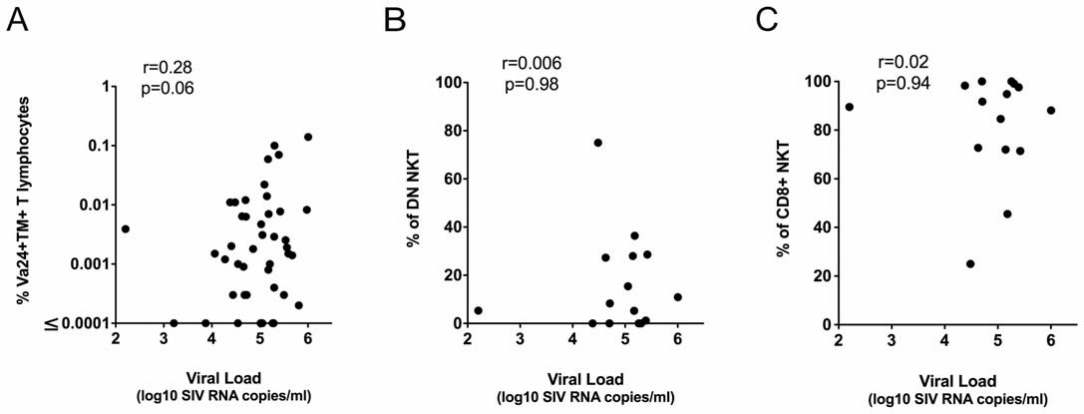
Relationship between plasma SIV RNA and NKT lymphocytes. (A) Correlation between plasma SIV RNA and peripheral blood frequency of Vα24^+^CD1d TM^+^ NKT lymphocytes in 43 SIV-infected sooty mangabeys. (B) Percentage of DN NKT lymphocytes and (C) percentage of CD8^+^ NKT lymphocytes correlated with plasma SIV RNA in 15 SIV-infected sooty mangabeys. ‘r’ denotes correlation coefficient values determined by the Pearson correlation test.

Previous studies have shown that CD4^+^ NKT lymphocytes are highly susceptible to *in vitro* HIV and SIV infection [43], [44], [45]. Selective depletion of peripheral blood NKT lymphocytes, not exclusively confined to CD4^+^ NKT cells, has been reported in HIV-infected humans [43], [46], [47] and SIV-infected pig-tailed macaques [54]. To assess if NKT lymphocytes are similarly depleted in sooty mangabeys with chronic SIV infection, we performed a cross-sectional analysis of the frequency of peripheral blood NKT lymphocytes in 50 SIV-negative and 50 naturally SIV-infected sooty mangabeys (Figure 8A–C). The median age of the SIV-negative sooty mangabeys was 11 years (range 4 to 20 years), while the median age of the SIV-infected mangabeys was 17 years (range 10 to 24 years). NKT lymphocyte frequencies >0.002% of circulating T lymphocytes were detected in 44% of SIV-negative sooty mangabeys (mean 0.007%) and in 52% of SIV-infected sooty mangabeys (mean 0.01%) and did not differ between the two groups (Figure 8A). The frequency of NKT lymphocytes within the CD8^+^ and DN T lymphocyte subsets were also similar between SIV-negative and naturally SIV-infected sooty mangabeys (Figure 8B–C), indicating that there was no depletion of CD4^−^ NKT lymphocytes in SIV-infected sooty mangabeys. Consistent with these findings, we did not detect a difference in the proportion of CD8^+^ and DN NKT lymphocytes between SIV-negative and SIV-infected sooty mangabeys (Figure 8D). These data suggest that neither the frequency nor the phenotype of NKT lymphocytes are perturbed in sooty mangabeys with long-standing SIV infection. NKT lymphocytes in SIV-infected sooty mangabeys appear to be functionally intact as evidenced by comparable levels of IFN-γ production in SIV-infected and SIV-negative mangabeys following NKT ligand-specific stimulation (Figure 8E). Finally, we investigated the relationship between plasma SIV viremia and the frequency of NKT lymphocytes in SIV-infected sooty mangabeys (Figure 9A–C). Unlike pathogenic SIV infection where an inverse correlation between viral load and the frequency of total and CD4^+^ NKT lymphocytes was observed [54], we did not detect a similar correlation in sooty mangabeys (Figure 9A–C). A trend for a positive correlation between plasma SIV RNA and the total frequency of circulating invariant NKT lymphocytes did not reach statistical significance (Figure 9A). Moreover, the percentage of DN and CD8+ NKT lymphocytes showed no correlation with viral load (Figure 9B–C).

## Discussion

In this study we present results of a comprehensive analysis of the phenotype and function of *ex vivo* and clonal NKT lymphocytes in sooty mangabeys. To our knowledge this is the first report of NKT cells in a natural nonhuman primate host of the simian immunodeficiency virus. The mechanism of chronic immune activation in pathogenic lentiviral infection remains a central unresolved question of AIDS pathogenesis. We reasoned that, in light of their immunoregulatory function, NKT cells have the potential to play a role in modulating immune activation during HIV and SIV infection. Thus, a study of this unique T lymphocyte subset in natural hosts might provide useful insight into differences between pathogenic and nonpathogenic lentiviral infection. We show that sooty mangabey NKT lymphocytes are a functionally diverse population that rapidly produces a broad array of Th1 and Th2 cytokines, undergo degranulation, and proliferate when stimulated with α-GalCer presented on CD1d molecules indicating both effector and immunoregulatory functional capabilities. NKT lymphocytes were potent responders since these functional properties were apparent even on short-term stimulation of *ex vivo* PBMC, where NKT lymphocytes accounted for <1% of T lymphocytes. Circulating NKT lymphocytes in sooty mangabeys were characterized by a unique absence of CD4^+^ T lymphocytes, a feature that distinguished them from mice, humans, and Asian macaques. Consistent with this observation, NKT lymphocytes were neither depleted nor reduced in frequency in naturally SIV-infected sooty mangabeys and appeared to be functionally intact. Our data indicate that sooty mangabey NKT cells are resistant to SIV infection in vivo. Whether persistence of functionally intact systemic NKT cells helps to down-modulate and maintain low levels of immune activation in nonpathogenic natural SIV infection remains to be determined.

NKT lymphocytes comprise a small fraction of peripheral blood T lymphocytes in mice (mean 1.5%±0.8) and an even smaller fraction in humans (range 0.01 to 1%) [43], [70]. Published reports of NKT lymphocytes in nonhuman primates have been confined to three AIDS-susceptible Asian macaque species, namely, cynomolgus macaques, pig-tailed macaques, and rhesus macaques [45], [54], [62], [63], [69]. *Ex vivo* peripheral blood NKT lymphocyte frequencies averaging 0.4% in rhesus macaques and 0.19% in pig-tail macaques have been reported [54], [63]. While the frequencies of circulating NKT lymphocytes in sooty mangabeys were lower than those reported in SIV-negative Asian macaques, important phenotypic differences were noted. NKT cells in mice and humans include two major sub-populations: CD4^+^ and DN T lymphocytes [21], [70]. NKT cells in macaques include varying proportions of CD8^+^ and CD4^+^ T lymphocyte subsets but have a striking absence of DN T lymphocytes [45], [54], [62], [63]. Sooty mangabey NKT cells were phenotypically distinct from murine and human NKT cells, but similar to cynomolgus and rhesus macaques in having a dominance of CD8^+^ NKT lymphocytes. However, in contrast to macaques but similar to mice and humans, mangabey NKT cells contained DN T lymphocytes which accounted for as high as 74% of circulating NKT lymphocytes (mean 21%). The unique feature of circulating NKT lymphocytes in sooty mangabeys which distinguished them from mice, humans, and all three Asian macaque species, was the striking absence of CD4^+^ T lymphocytes. CD4^+^ single-positive or double-positive (CD4^+^CD8^+^) NKT lymphocytes account for approximately 50% of circulating NKT lymphocytes in humans [61], [68] and 20–80% of NKT lymphocytes in macaques [45], [54]. Similar to murine and human NKT lymphocytes, sooty mangabey NKT lymphocytes expressed high levels of NKG2D and moderate to high levels of CD161 [71], [72], [73], [74]. The effector memory phenotype and dominant expression of CXCR3 on *ex vivo* NKT cells in sooty mangabeys suggested that similar to humans, they had the ability to traffic to extra-lymphoid sites of inflammation [66], [75]. Unlike humans and macaques, the majority of NKT cells in sooty mangabeys did not express surface CCR5. *In vitro* experiments have shown that human and macaque CD4^+^ NKT lymphocytes are preferred targets of HIV and SIV infection in which the viruses replicate more rapidly in comparison to conventional CD4^+^ T lymphocytes [43], [45]. The lack of CD4 and CCR5 on sooty mangabey NKT cells suggests that they are not susceptible to SIV infection, despite recruitment to inflammatory sites.

Is there a functional consequence to the lack of CD4^+^ NKT cells in sooty mangabeys? Studies in humans have shown that DN NKT cells mainly produce Th1 cytokines while the CD4^+^ NKT cells produce both Th1 and Th2 cytokines [61], [68]. However, despite these differences, a recent study reported that human CD4^+^ NKT lymphocyte subsets preferentially induced Th1 responses, whereas the DN subset induced a shift toward Th2 responses through NKT-DC cross-talk along with elimination of IL-12-producing DC by direct NKT cytolysis [76]. Thus the association between phenotype and cytokine-secreting profile of NKT lymphocytes is not necessarily a simple one. Despite the absence of CD4^+^ NKT cells, *ex vivo* NKT lymphocytes and NKT cell clones in sooty mangabeys produced a wide array of Th1 and Th2 cytokines, including IL-10. At the single-cell level, Th2 cytokine secretion was mediated by a heterogeneous population of NKT cells (Figure 5D). Thus, IL-13 was produced by both IFN-γ-secreting and non-IFN-γ-secreting NKT cells. Moreover, sooty mangabey DN NKT lymphocytes produced both Th1 and Th2 cytokines. These observations reinforce the concept that functional differences in NKT lymphocytes are not solely associated with their CD4/CD8 phenotype. Other factors, such as the cytokine microenvironment and NKT-APC interaction are likely to determine the Th1/Th2 bias of a responding NKT lymphocyte.

The limited published data on functionality of NKT lymphocytes in AIDS-susceptible macaque species does not allow for a complete comparative analysis of the function of sooty mangabey and macaque NKT lymphocytes. In one study on long-term cultured splenic NKT lymphocytes in rhesus macaques, cytokine secretion in response to α-GalCer stimulation was dominated by TGF-β and IL-13 production, with low levels of IFN-γ and little or no IL-2, IL-4 or IL-10 secretion, although IL-10 was produced intracellularly [77]. In contrast, we observed that sooty mangabey NKT clones readily produced high levels of IFN-γ along with IL-2, IL-4, IL-10, and IL-13 on α-GalCer stimulation. Moreover, with the exception of IL-4, the same pattern of cytokine secretion was also observed on α-GalCer stimulation of *ex vivo* PBMC that contained low frequencies of NKT lymphocytes. IFN-γ and IL-2 secretion was apparent as early as two hours following α-GalCer stimulation of *ex vivo* PBMC. Early IFN-γ secretion by NKT lymphocytes might help in recruitment and activation of downstream effectors such as NK cells and CD8^+^ T lymphocytes, as well as enhance DC maturation, thereby promoting immune responses to pathogens. Studies in mice have demonstrated efficient induction of CD8^+^ T cell responses by NKT lymphocytes through early IFN-γ production during acute virus infection [33], [38], [78]. IL-2, on the other hand, is a primary growth factor for antigen-activated T lymphocytes and also acts as the major inducer for the development of suppressive Treg cells. Indeed, activation of NKT lymphocytes with α-GalCer has been demonstrated to enhance IL-2 production by NKT lymphocytes and subsequent expansion of Tregs [79], [80]. In this regard it is interesting that a recent comparative study of acute SIV infection in African green monkeys (AGM) and pig-tailed macaques showed an earlier increase of Tregs in AGM that correlated with resolution of immune activation [15]. Likewise, IL-4 and IL-10 produced by NKT lymphocytes can induce Treg cells [81], and IL-13 can act on myeloid cells to induce TGF-β [82]. Thus, the broad array of cytokines produced by sooty mangabey NKT lymphocytes indicate that they have the capability to mediate both effector functions and suppress immunopathology. The potent production of IL-10 by sooty mangabey NKT lymphocytes, a feature notably absent in rhesus macaques [77], suggests that there may be species-specific functional differences in NKT lymphocytes. In light of these findings, it is tempting to speculate that the presence of functionally intact NKT cells in SIV-infected sooty mangabeys could help to blunt non-specific immune activation in sooty mangabeys as opposed to rhesus macaques. While the low levels of circulating invariant NKT lymphocytes in sooty mangabeys raises questions about their overall impact on systemic immune activation, it is possible that the presence of higher frequencies of tissue-resident NKT cells at mucosal effector sites could help blunt hyper-immune activation.

In summary, we have characterized the phenotype and function of sooty mangabey NKT cells and demonstrated that they are a heterogeneous population comprised chiefly of CD8^+^ and DN T lymphocyte subsets with a CXCR3-positive memory phenotype, and expression of NKG2D and CD161 as the prominent NK cell markers. We show that CD1d-restricted sooty mangabey NKT cells are multifunctional and resistant to SIV infection. By virtue of their cytolytic and Th1 cytokine-secreting ability, combined with IL-10 production, it is possible that sooty mangabey NKT cells facilitate early induction of an appropriate antiviral immune response while concurrently dampening non-specific chronic immune activation. Further studies are required to investigate the role of these T cell subsets in modulation of immune responses in pathogenic and nonpathogenic SIV infection.

## Materials and Methods

### Ethics Statement

All animals were maintained in accordance with Emory University's Institutional Animal Care and Use Committee and federal guidelines for animal care.

### Animals

Blood samples used in this study were obtained from fifty SIV-uninfected and fifty naturally SIV-infected sooty mangabeys. All animals were housed at the Yerkes National Primate Research Center (YNPRC), Atlanta.

### Sample collection

Sooty mangabey blood was collected at YNPRC in heparin vacutainer tubes (Becton Dickinson Vacutainer systems, Franklin Lakes, NJ), and shipped overnight on ice to the New England Primate Research Center (NEPRC) where it was processed the following day. Peripheral blood mononuclear cells (PBMC) were separated by density gradient centrifugation (Lymphocyte Separation Medium; MP Biomedicals Inc., Solon, OH) at 1500 rpm for 45 minutes and used for phenotyping and *in vitro* assays.

### Immunophenotyping and flow cytometry of NKT lymphocytes

Multicolor flow cytometric analysis was performed on *ex vivo* and *in vitro* expanded cells according to standard procedures using anti-human mAbs that cross-react with sooty mangabeys. NKT lymphocyte ligand PBS-57-loaded and unloaded human CD1d Tetramers (CD1d TM) conjugated with APC were obtained from the NIH Tetramer core facility. The following antibodies were obtained from BD Biosciences unless stated otherwise: anti-Vα24–PE (clone C15; Immunotech), 6B11–FITC (6B11), anti-CD3–APC-Cy7 (SP34-2), anti-CD4–Qdot605 (T4/19Thy5D7; custom/NHP Resource), anti-CD8–Alexa Fluor 700 (RPA-T8), anti-CD56–PE-Cy7 (NCAM16.2), anti-CD16–Alexa Fluor 700 (3G8; Invitrogen), anti-CD161–APC (DX12), anti-NKG2D–PE (ON72; Beckman Coulter), anti-CD95–PE-Cy5 (DX2), anti-CD28–PE TexasRed (CD28.2; Immunotech), anti-CD45RA–PE-Cy7 (L48), anti-CCR7–biotin (150503; custom), anti-CCR5–APC (3A9), anti-CXCR3–PE (1C6), anti-CD69–PE TexasRed (TP1.55.3; Beckman Coulter), anti-CD107a–PE (H4A3), anti-Perforin–FITC (B56), anti-GranzymeB–APC (GB12; Caltag), anti-IFN-γ–PE-Cy7 (B27), anti-IL-2–APC (MQ1-17H12), anti-IL-13–FITC (PVM13-1; eBioscience), anti-TNF-α–Alexa Fluor 700 (MAb11).

For identification of NKT cells, PBMCs were surface stained for CD3 and anti-Vα24 combined with PBS-57 loaded CD1d TM or 6B11 antibody. APC-labeled unloaded CD1d TM controls were used in all experiments. Surface staining was carried out by standard procedures. Briefly, 2 to 4 million PBMC re-suspended in 100 µl wash buffer (PBS with 2% FBS) were initially incubated with tetramers for 20 min at 4°C followed by addition of surface antibodies and further incubation for 30 min at 4°C. After washing, the cells were fixed in 2% paraformaldehyde. All intracellular cytokine staining (ICS) assays were carried out on cells that were stimulated overnight. Following 16 h incubation, cells were washed in PBS containing 2% FCS and 0.5 mM EDTA, stained for surface markers in wash buffer for 30 min at 4°C, washed and then fixed and permeabilized using the Invitrogen Fix/Perm reagents (CALTAG™). Permeabilized cells were stained intracellularly with the requisite antibodies. Cells were then washed in wash buffer and fixed in 2% paraformaldehyde. Flow cytometric acquisition was performed on an LSR-II cytometer driven by the FACS DiVa software (version 5.2; BD). At least 200,000 T lymphocyte events were collected. Analysis of the acquired data was performed using FlowJo software (version 8.8.3; TreeStar, Ashland, OR). For experiments measuring three-functional responses, Boolean gating was used to partition cells into specific response categories and PESTLE v1.6.2 (provided kindly by Dr Mario Roederer) and SPICE 5.05 software (provided free of charge by NIAID/NIH in collaboration with Dr. Mario Roederer) was used to analyze data.

### Medium and Reagents

The complete medium (R10 medium) used throughout was RPMI medium 1640 (Cellgro, Herndon, VA) supplemented with 10% FCS (Sigma-Aldrich, St. Louis, MO), 1% 1 M HEPES, 2 mM L-glutamine (Cellgro), 50 IU/ml penicillin (Cellgro), 50 µg/ml streptomycin (Cellgro). The NKT-ligand α-galactosylceramide (α-GalCer, Diagnocine LLC, Hackensack, NJ) was used at 100 ng/ml. The anti-human CD1d antibody (Clone 42.1) described earlier [83] was used at 0.1–20 µg/ml. Recombinant human IL-2 (Roche) was used at 10–50 IU/ml of medium for the expansion and maintenance of NKT cell clones.

### In vitro expansion of NKT lymphocytes

6B11-positive lymphocytes were sorted on a FACSAria cell sorter (BD Biosciences, San Jose, CA, USA) and cloned by limiting dilution at 3 and 10 cells per well in 96-well, round-bottom, polystyrene plates (Corning, NY, USA). Cells were incubated in R10 medium containing 5 µg/ml ConA and 100 ng/ml of α-GalCer along with 100,000 cells/well of human feeder PBMC irradiated at 3000 rads. After two days, ConA was removed and 50 IU/ml recombinant human IL-2 was added. Wells with cell outgrowth were re-stimulated and expanded over a 2–4 week period. The presence of NKT clones was confirmed by staining with anti-Vα24, PBS-57 loaded CD1dTM or 6B11 mAb. Positive NKT clones were maintained in complete media supplemented with 50 IU/ml IL-2. For functional analyses, the clones were gradually switched to resting stage (10 IU/ml IL-2) 48 h prior to the assay as previously described [84].

### Functional Analysis of NKT lymphocytes

For NKT cell activation assays, 2×10^4^ NKT cells or 10^5^ PBMCs were plated in a 96-well flat-bottom plate with either medium alone or with an equal number of stimulator cells that had been treated with α-GalCer at a final concentration of 100 ng/ml. 25 ng/ml PMA (Sigma-Aldrich, St. Louis, MO) with 1 µg/ml calcium ionophore (PMA/Ca) was used as positive control stimulus. 50,000 CD1d-transfected C1R B cell line (C1R.d) were γ-irradiated at 10,000 rads and used as APCs for the presentation of α-GalCer as previously described [67]. Irradiated mock-transfected C1R cells served as a negative control stimulus for NKT cells. For stimulation of clones with CD1d transfectants, IL-2 was included in the culture medium at a final concentration of 10 IU/ml. Typically, proliferation and cytokine release assays on NKT cell clones were performed 3–4 wk after the last re-stimulation. All measurements were performed in triplicate.

### CFSE proliferation assay

NKT cells were re-suspended at a concentration of ≤5×10^6^/ml in RPMI 1640 (Cellgro, Herndon, VA) and labeled with 2 µM CFSE (Molecular Probes, Eugene, OR) by incubation for 10 min at 37°C in a 5% CO_2_ incubator. The CFSE label was quenched with addition of 1x volume of 100% FBS. After incubation for 1 min at RT, the dye was diluted further with RPMI- 1640 and the cells were washed twice before culturing in flat-bottom 96-well plates. FACS analysis was performed after 5 days of incubation.

### Statistical Analysis

Paired and unpaired *t*-tests were used for comparisons of grouped and ungrouped samples respectively. The Pearson test was performed for correlation analysis. *P*<0.05 was considered statistically significant. All statistical analyses were performed using the GraphPad Prism software version 5.0b (GraphPad Software, Inc., La Jolla, CA).
